# TLR Agonists Modify NK Cell Activation and Increase Its Cytotoxicity in Acute Lymphoblastic Leukemia

**DOI:** 10.3390/ijms25137500

**Published:** 2024-07-08

**Authors:** Janet Gallardo-Zapata, Erandi Pérez-Figueroa, Víctor Olivar-López, Aurora Medina-Sansón, Elva Jiménez-Hernández, Enrique Ortega, Carmen Maldonado-Bernal

**Affiliations:** 1 Immunology and Proteomics Research Laboratory, Hospital Infantil de México Federico Gómez, Mexico City 06720, Mexico; 2 Faculty of Medicine, Universidad Nacional Autónoma de México, Mexico City 04360, Mexico; 3 Emergency Service, Hospital Infantil de México Federico Gómez, Mexico City 06720, Mexico; 4 Hemato-Oncology Department, Hospital Infantil de México Federico Gómez, Mexico City 06720, Mexico; auroramedina@aol.com.mx; 5 Hematology Service, Hospital Pediátrico Moctezuma, Mexico City 15530, Mexico; 6 Department of Immunology, Institute of Biomedical Research, Universidad Nacional Autónoma de México, Mexico City 4510, Mexico

**Keywords:** natural killer cells, toll-like receptors, cytotoxicity, acute lymphoblastic leukemia, IFN-γ, NKG2D, NKp44

## Abstract

Natural killer (NK) cells play a crucial role in innate immunity, particularly in combating infections and tumors. However, in hematological cancers, NK cells often exhibit impaired functions. Therefore, it is very important to activate its endosomal Toll-like receptors (TLRs) as a potential strategy to restore its antitumor activity. We stimulated NK cells from the peripheral blood mononuclear cells from children with acute lymphoblastic leukemia and NK cells isolated, and the NK cells were stimulated with specific TLR ligands (Poly I:C, Imiquimod, R848, and ODN2006) and we evaluated changes in IFN-γ, CD107a, NKG2D, NKp44 expression, Granzyme B secretion, cytokine/chemokine release, and cytotoxic activity. Results revealed that Poly I:C and Imiquimod enhanced the activation of both immunoregulatory and cytotoxic NK cells, increasing IFN-γ, CD107a, NKG2D, and NKp44 expression. R848 activated immunoregulatory NK cells, while ODN2006 boosted CD107a, NKp44, NKG2D, and IFN-γ secretion in cytotoxic NK cells. R848 also increased the secretion of seven cytokines/chemokines. Importantly, R848 and ODN 2006 significantly improved cytotoxicity against leukemic cells. Overall, TLR stimulation enhances NK cell activation, suggesting TLR8 (R848) and TLR9 (ODN 2006) ligands as promising candidates for antitumor immunotherapy.

## 1. Introduction

Natural Killer (NK) cells represent a highly specialized subpopulation of lymphoid cells (5–15%), integral to the innate immune system [1]. These cells provide early immunoregulatory cytokines and tumor cell lysis without previous stimulation, crucial for immunological surveillance and tumor suppression. The surface antigens CD56 and CD16 antigens allow the characterization of NK cells and categorize them as regulatory and cytotoxic, depending on their expression levels [2].

Approximately 90% of human NK cells in peripheral blood are cytotoxic, with low expression of CD56 (CD56dim/−) and high levels of the Fcγ receptor III (FcγRIII, CD16bright), while 10% are immunoregulatory, which are CD56brightCD16dim or CD16−. Of these two subpopulations, CD56dimCD16bright cells have greater cytotoxic activity, while CD56brightCD16dim cells favor the synthesis of inflammatory cytokines [2,3].

NK cells express numerous receptors on their membrane, which mediate different signals for the activation of their immune activity against infections and tumor cells. Since NK cells do not have clonal receptors, their repertoire is composed of germline receptors, which have traditionally been classified as activating receptors (triggering the function of the NK cells) and inhibitory receptors (those that regulate the signal delivered by activating receptors and prevent NK cell activation) [2]. In addition, NK cells express pattern recognition receptors (PRRs), such as Toll-like receptors, through which they recognize pathogen-associated molecular patterns (PAMPs) as well as damage-associated molecular patterns (DAMPs) [4].

Acute lymphoblastic leukemia (ALL) constitutes 80% of all leukemias in children [5]. Even though the survival of patients with ALL has increased remarkably, 20% of patients are still resistant to treatment [6]. NK cells play a central role in identifying and eradicating leukemia cells [7,8]; however, studies have reported that peripheral blood NK cells exhibit compromised cytotoxicity towards autologous blasts [9,10] with altered cell surface expression of inhibitory receptors (NKp46 and NKG2A) compared to age-matched healthy control subjects [11,12]. Therefore, the discovery that TLRs are also expressed in NK cells has increased the interest in their immune response against tumor cells.

TLRs constitute a family of transmembrane proteins, that recognize specific molecular patterns from microorganisms, as well as endogenous ligands that produce inflammatory responses [13]. Of the 11 TLRs recognized in humans, 10 are expressed as proteins, with TLRs 1, 2, 4, 5, 6, and 10 expressed on the cell membrane and recognizing mainly microbial components such as LPS, proteins, and lipoproteins [14], while TLRs 3, 7, 8, and 9 are mainly expressed in endosomes where they recognize nucleic acid components such as double-stranded RNA (dsRNA), single-stranded RNA (ssRNA), and unmethylated CpG DNA motifs [15,16,17]. TLR11 is considered a pseudogene [18]. The binding of TLRs to their corresponding ligand triggers a signaling pathway leading to the activation of the immune response for the elimination of pathogens [19,20,21].

It has been described that NK cells express endosomal TLRs [22]; however, even though there is evidence of the direct activation of NK cell functions through these TLRs, this activation was evaluated in healthy donor NK cells and the researchers also discovered that this can only occur by the complex interaction with other cells of the immune system and the microenvironment [23,24,25,26,27]. Although different preclinical studies have shown the effective activation of donor NK cells with TLR ligands to induce blast lysis [28,29,30], the effects of this activation in the context of hematological disorders, such as ALL, remains unknown [31]. Since NK cells are important for effective tumor suppression demonstrating that the activation of these cells from hematological oncological disorders is possible, would provide a new panorama for the development of antitumoral therapies.

Previously in our work group, Sánchez et al. demonstrated that the NK cells from ALL pediatric patients (bone marrow and peripheral blood), express all the endosomal TLRs (TLR3, TLR7, TLR8 and TLR9) [22]. Therefore, the aim of this study was to evaluate the response of the different subpopulations of human NK cells from pediatric ALL patients to stimulation through their different endosomal TLRs using synthetic TLR agonists and compare this response with those from unstimulated human NK cells from pediatric patients with ALL in order to evaluate the feasibility of inducing NK cell activation as a first approach as a possible adjuvant in antitumor immunotherapy.

## 2. Results

### 2.1. Characteristics of the Patients

Peripheral blood NK cells from patients recently diagnosed with ALL without treatment, transfusion, and/or infection were analyzed. There were 14 boys and 10 girls between the ages of 1 and 17 years old. Ten patients were used for each determination, depending on the number of cells that could be purified from each patient. The characteristics of the study group are summarized in Table 1.

### 2.2. Identification of the Different NK Cell Subpopulations in Patients with ALL and IFN-γ, CD107a, NKp44, and NKG2D Expression

**Figure 1:**
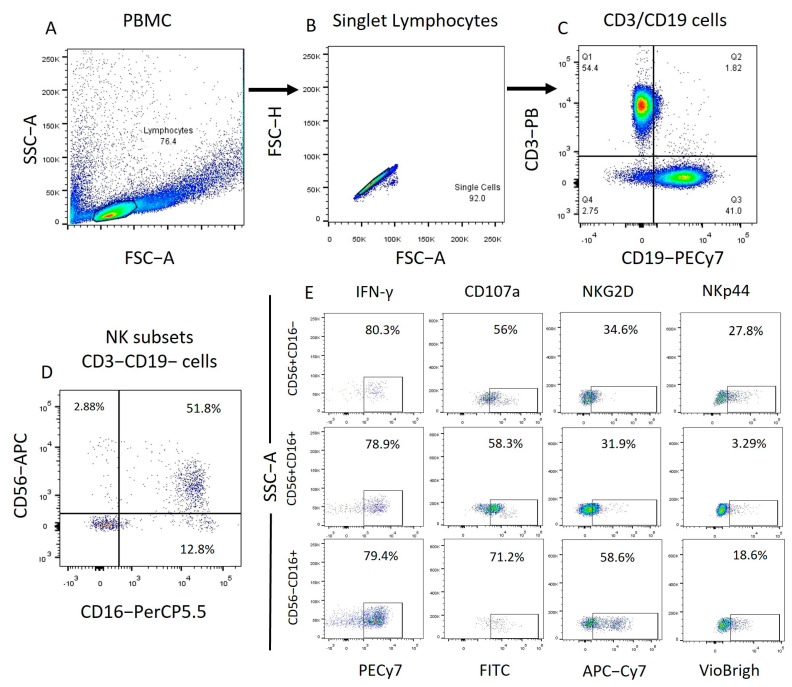
PBMC Flow cytometry analysis strategy. We obtained the total NK cells (CD3−, CD19−) from the lymphocyte population identified by granularity size and from this population we obtained the NK cells subpopulations depending on their expression of CD56 and CD16. Finally, from each subpopulation, we obtained the expression of the markers to be analyzed.

**Figure 2:**
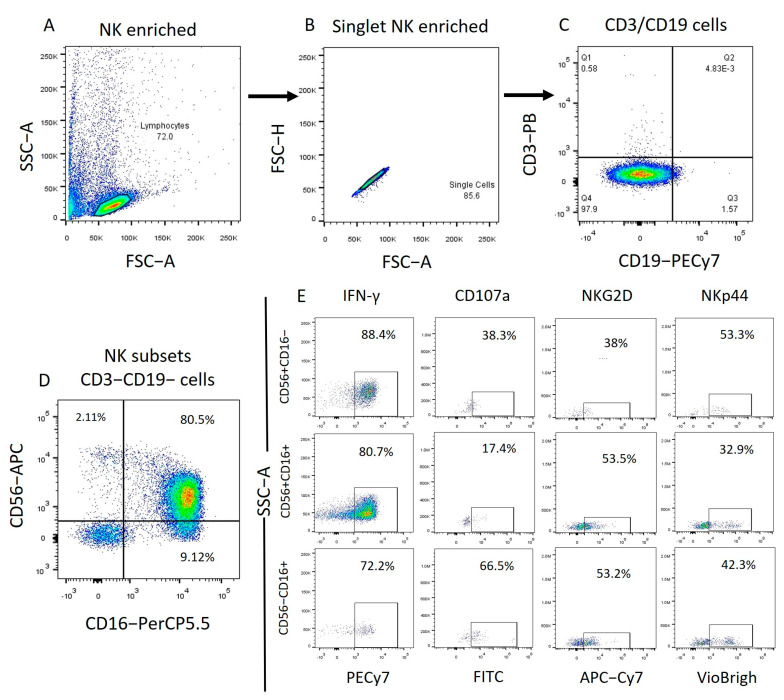
Enriched NK cells Flow cytometry analysis strategy. We obtained the total NK cells (CD3−, CD19−) from the lymphocyte population identified by granularity size and from this population we obtained the NK cell subpopulations depending on their expression of CD56 and CD16. Finally, from each subpopulation, we obtained the expression of the markers to be analyzed.

The different NK cell subpopulations of all samples were characterized in both whole PBMCs and enriched NK cells. After enrichment, the samples displayed more than 96% NK cells. NK cells were characterized by selecting cells with CD56/CD16 expression from the previously gated CD3−CD19− subpopulation. Within the NK cell populations of all samples, immunoregulatory cells (CD56+, CD16−) and cytotoxic cells (CD56+, CD16+ and CD56−, CD16+) were identified. For each NK cell subpopulation, the mean fluorescence intensities and percentages of alive positive cells were determined for key markers such as: CD107a, IFN-γ, NKG2D, and NKp44 (Figure 1, Figure 2, Figures S1 and S2).

### 2.3. Poly I:C and R848 Increase the Activity of NK Subpopulations within the PBMC

To assess the impact of TLR ligands on the function of NK cell subpopulations within mononuclear cells, we cultured them for 24 h with R848 (TLR7/8 ligand), Poly I:C (TLR3 ligand), Imiquimod (TLR7 ligand), or ODN2006 (TLR9 ligand) and analyzed them for IFN-γ production and CD107a expression.

**Figure 3:**
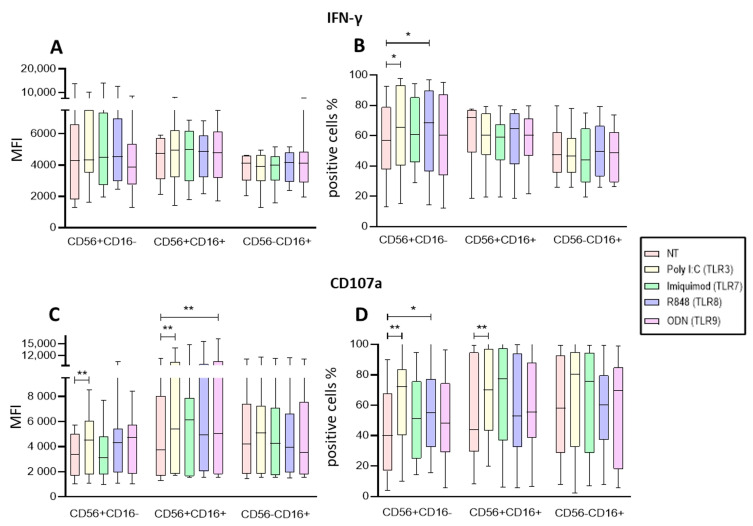
Functional Activity of the NK cells within PBMCs after endosomal TLR stimulation. (**A**) The MFI of intracellular IFN-γ was determined in the three subpopulations for each treatment and compared to the MFI of non-treated cells. (**B**) The median of the percentage of the IFN-γ expressing cells was determined and compared with the percentage of non-treated cells expressing IFN-γ. (**C**) The median of the Median Fluorescence Intensity (MFI) of CD107a was determined for each treatment in the three subpopulations and compared to the MFI of non-treated cells (control). (**D**) The median of the percentage of the CD107a-expressing cells was determined for each treatment in the three subpopulations and compared with the percentage of non-treated cells expressing CD107a. The box and whisker plot was employed to visualize the distribution of the data, highlighting the central tendency, spread and the outlier’s values within the dataset. The Friedman test was used for the comparison. * *p* < 0.05, ** *p* < 0.005.

When the regulatory activity of the NK subpopulations after stimulation within the PBMC fraction was evaluated, none of the treatments modified the production of intracellular IFN-γ among the three subpopulations, (Figure 3A). However, the proportions of IFN-γ+ cells significantly increased after the stimulation with Poly I:C (*p* = 0.0187) and R848 (*p* = 0.0288) in the immunoregulatory subpopulation CD56+CD16− (Figure 3B).

Subsequently, the degranulation of NK cells was evaluated by the expression of CD107a, a degranulation marker. We observed a significant increase in the CD107a expression on CD56+CD16− (*p* = 0.0075) after stimulation with Poly I:C (*p* = 0.0075) and on CD56+CD16+ after Poly I:C (*p* = 0.0075), and ODN2006 (*p* = 0.0075) stimulation (Figure 3C). When analyzing the proportions of cells expressing CD107a, we found that Poly I:C significantly increased the proportion of CD56+CD16− and in the CD56+CD16+ subpopulation (*p* = 0.0036 and *p* = 0.0012, respectively). Additionally, R848 increased the proportion of the immunoregulatory subpopulation (CD56+CD16−) (*p* = 0.0288) (Figure 3D).

Poly I:C and R848 seem to have an effect on the immunoregulatory NK subsets, while ODN influences more the Cytotoxic NK subset, within the contexts of PBMCs.

### 2.4. Imiquimod and ODN2006 Increase the Function of Enriched NK Cells Subpopulations

**Figure 4:**
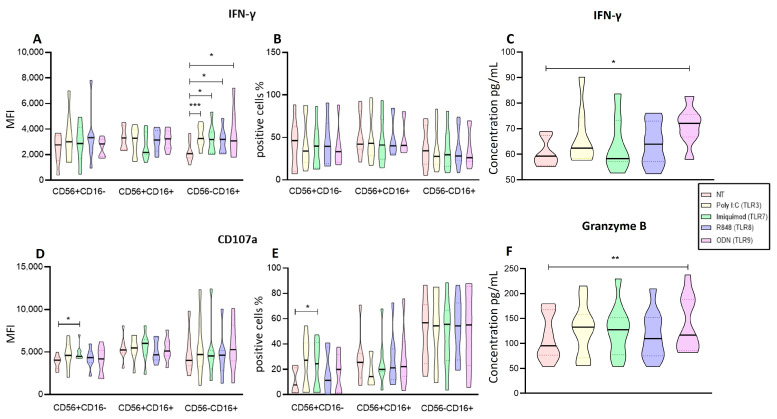
Functional Activity of the enriched NK cells after endosomal TLR stimulation. (**A**) The MFI of IFN-γ was determined for each treatment in the three subpopulations and compared to the non-treated MFI. (**B**) The median of the percentage of the IFN-γ expressing cells was determined and compared with the percentage of non-treated cells expressing IFN-γ. (**C**) Concentration of IFN-γ in supernatants of enriched NK cells after endosomal TLR stimulation. (**D**) The median of the Median Fluorescence Intensity (MFI) of CD107a was determined for each treatment in the three subpopulations and compared to the MFI of non-treated cells (control). (**E**) The median of the percentage of the CD107a-expressing cells was determined and compared with the percentage of non-treated cells expressing CD107a. (**F**) The concentration of Granzyme B in supernatants of enriched NK cells after endosomal TLR stimulation. The violin plot was employed similar to the box-and-whiskers plot, to visualize the full distribution of the data, providing shape and density within the dataset. The Friedman test was used for the comparison. * *p* < 0.05, ** *p* < 0.005, *** *p* < 0.0005.

To further assess the specific response of the TLR activation on the NK cells, we stimulated enriched NK cells (purity 96.3%) with Poly I:C, Imiquimod, R848, and ODN2006. The mean fluorescence intensities and the percentages of cells expressing the different markers, CD107a, IFN-γ, NKG2D, and NKp44, were determined for each subpopulation and secretion of Granzyme B and IFN-γ was evaluated in the supernatants (Figure 4).

The immunoregulatory activity was evaluated by measuring the intracellular production of IFN-γ. In this case, only the cytotoxic subpopulation (CD56−CD16+) significantly increased the expression of IFN-γ (Poly I:C *p* < 0.005, Imiquimod, R848 and ODN, *p* < 0.05) with the four treatments (Figure 4A). When the proportions of IFN-γ+ cells were analyzed, we observed that the three subpopulations remained without significant change in proportions after the four treatments (Figure 4B).

Additionally, we measured the concentration of IFN-γ (pg/mL) in the supernatant of enriched NK cells cultured with the four different ligands (Figure 4C). We found that ODN significantly increased the production of IFN-γ (*p* = 0.0352) compared to untreated cells.

When we assessed the degranulation of the NK cells by measuring the expression of CD107a, only the immunoregulatory (CD56+CD16−) subpopulation increased the CD107a expression significantly when stimulated with Imiquimod (*p* = 0.0449) as compared with the non-treated cells (Figure 4D). Imiquimod also significantly increased the proportion of cells expressing this marker (*p* = 0.0162) in the immunoregulatory subpopulation (CD56+CD16−) (Figure 4E).

To further evaluate degranulation, we evaluated the concentration of granzyme B by enriched NK cells cultured with the four different ligands (Figure 4F). We observed that ODN significantly increased the secretion of Granzyme B (*p* = 0.0075).

**Figure 5:**
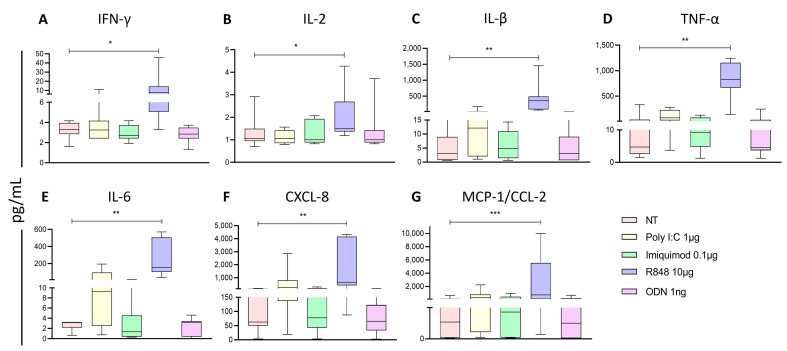
Proinflammatory cytokines in PBMC supernatants after endosomal TLR stimulation. (**A**) The concentration of IFN-γ, (**B**) IL-1β, (**C**) IL-2, (**D**) TNF-α, (**E**) IL-6, (**F**) IL-8, and (**G**) MCP-1 was determined for each treatment and compared to the non-treated cells. The Friedman test was used for the comparison. * *p* < 0.05, ** *p* < 0.005, *** *p* < 0.0005.

In addition, to determine the increase in the immunoregulatory function, we measured the concentration of different proinflammatory cytokines and chemokines in the supernatant of the PBMC cultured with the four different ligands (Figure 5). We found that R848 induced a significant increase in secretion of IFN-γ (Figure 5A), IL-2 (Figure 5B), IL-1β (Figure 5C), TNF-α (Figure 5D), IL-6 (Figure 5E), IL-8/CXCL-8 (Figure 5F), and MCP-1/CCL-2 (Figure 5G), after 24 h of stimulation with this ligand compared with the non-treated cells.

### 2.5. Endosomal TLRs Increase the Activation of NK Subpopulations within the PBMC

**Figure 6:**
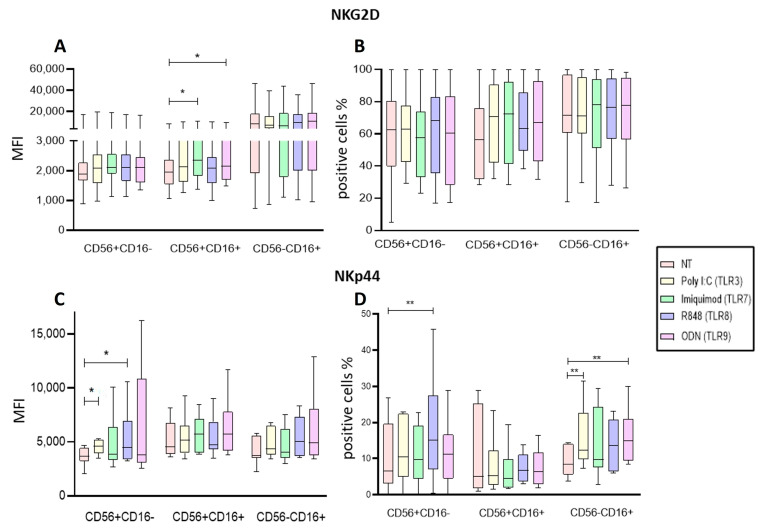
Analysis of Activation markers of the different subpopulations of NK cells from PBMCs after endosomal TLR stimulation. (**A**) The MFI of NKG2D expression was determined for each treatment in the three subpopulations and compared to the MFI of non-treated cells. (**B**) The median of the percentage of NKG2D-expressing cells after treatment with the TLRs ligands was determined and compared with the percentage of non-treated cells expressing it. (**C**) The MFI of NKp44 expression was determined for each treatment in the three subpopulations and compared to the MFI of non-treated cells. (**D**) The median of the percentage of NKp44-expressing cells after treatment with the TLR ligands was determined and compared with the percentage of non-treated cells expressing it. The box and whisker plot was employed to visualize the distribution of the data, highlighting the central tendency, spread and the outlier’s values within the dataset. The Friedman test was used for the comparison. * *p* < 0.05, ** *p* < 0.005.

To determine if the activation of NK cells from leukemia patients is dependent on their NK receptors (NCR), we evaluated the expression of NKG2D and NKp44 receptors in the different NK subpopulations by culturing PBMC for 24 h with Poly I:C, Imiquimod, R848 or ODN2006 (Figure 6).

Imiquimod (TLR7) and ODN (TLR9) significantly increased NKG2D expression in the cytotoxic CD56+CD16+ (*p* = 0.0356 and *p* = 0.0233, respectively) subpopulation (Figure 6A). When we analyzed the proportions of NKG2D-positive cells, we found that none of the treatments significantly increased the percentage of NKG2D-positive cells in any of the subpopulations (Figure 6B).

When the expression of NKp44 on the NK subpopulations was assessed, Poly I:C and R848 significantly increased this marker expression on the CD56+CD16− subpopulation (*p* = 0.0436 and *p* = 0.0436, respectively) (Figure 6C). Regarding the proportions of NKp44+ cells, R848 significantly increased the percentage of CD56+CD16− expressing NKp44 (*p* = 0.0016); CD56−CD16+ expressing NKp44 significantly increased when they were stimulated with Poly I:C (*p* = 0.0075) and ODN (*p* = 0.0094), (Figure 6D).

Our results show that NK activation can be increased when stimulated with the different TLR ligands within the PBMC context.

### 2.6. Endosomal TLR Ligands Increase the Activation of Enriched NK Cell Subpopulations

**Figure 7:**
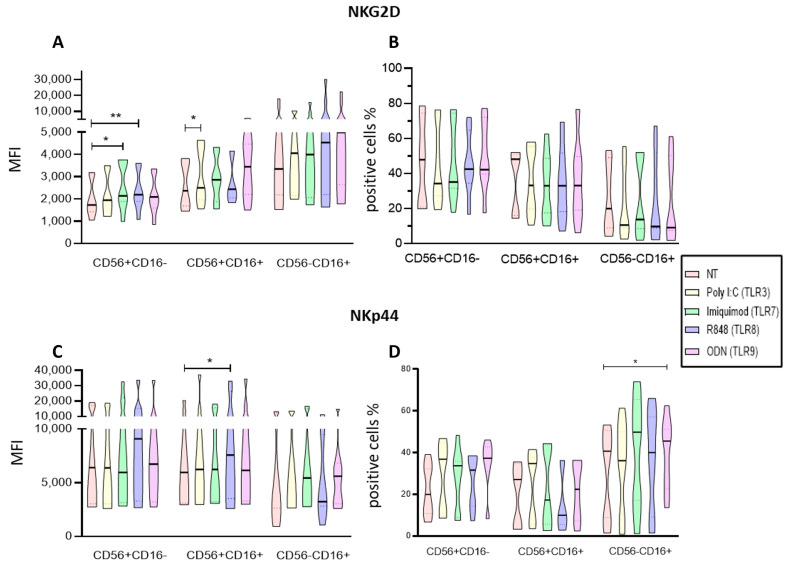
Analysis of Activation markers of the different subpopulations of enriched NK cells after endosomal TLR stimulation. (**A**) The MFI of NKG2D expression was determined for each treatment in the three subpopulations and compared to the MFI of non-treated cells. (**B**) The median of the percentage of NKG2D-expressing cells after treatment with the TLR ligands was determined and compared with the percentage of non-treated cells expressing it. (**C**) The MFI of NKp44 expression was determined for each treatment in the three subpopulations and compared to the MFI of non-treated cells. (**D**) The median of the percentage of NKp44-expressing cells after treatment with the TLR ligands was determined and compared with the percentage of non-treated cells expressing it. The violin plot was employed similar to the box-and-whiskers plot, to visualize the full distribution of the data, providing shape and density within the dataset. The Friedman test was used for the comparison. * *p* < 0.05, ** *p* < 0.005.

We then analyzed the expression of NKG2D and NKp44 receptors in the different enriched NK cell subpopulations after culturing with Poly I:C, Imiquimod, R848, or ODN2006 (Figure 7). We observed that Imiquimod and R848 significantly increased NKG2D expression in the immunoregulatory subpopulation (CD56+CD16−) (*p* = 0.0162 and *p* = 0.0094, respectively), while poly I:C significantly increased the NKG2D expression in the cytotoxic subpopulation CD56+CD16+ (*p* = 0.0352) compared with the non-treated cells. (Figure 7A). When analyzing the proportions of NKG2D-positive cells, we found that none of the treatments significantly modified it in any of the subpopulations (Figure 7B).

When we analyzed the expression of NKp44, we observed that only R848 significantly increased the expression of NKp44 on the CD56+CD16+ subpopulation (*p* = 0.0274) (Figure 7C). In the evaluation of the proportions of NKp44+ cells, we observed that ODN significantly increased the percentage of NKp44-positive cells for the cytotoxic subpopulation CD56−CD16+ (*p* = 0.0162) (Figure 7D).

Taking these results together, they suggest that the Poly I:C, R848 and OD2006 ligands (TLR3, TLR8, and TLR9, respectively) promote an activated status in the NK cells.

### 2.7. R848 Increase HLA-I Expression in Immature B Cells of Patients with ALL

**Figure 8:**
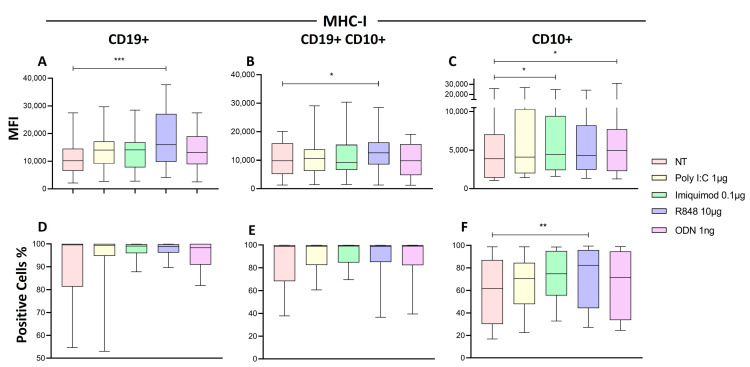
Expression of HLA-I in the lymphoblast from patients with ALL. (**A**,**D**) The CD19+ represents the mature B cells, (**B**,**E**) CD10+ represents the marker of lymphoid progenitor cells, and (**C**,**F**) CD10+ CD19+ represents the pre-B cells from the patients with ALL. The median of MFI (**A**–**C**) and the median of the percentage of HLA-I (**D**–**F**) were determined for each treatment and compared to the non-treated cells. The box and whisker plot was employed to visualize the distribution of the data, highlighting the central tendency, spread and the outlier’s values within the dataset. The Friedman test was used for the comparison. * *p* < 0.05, ** *p* < 0.005, *** *p* < 0.0005.

We determined if the stimulation with synthetic TLR ligands affects the density of HLA-I in the lymphoblastic cells in the patient’s blood. We observed that the expression of HLA-I on mature B cells (CD19+) (Figure 8A) and B cell precursors (CD19+CD10+) (Figure 8B) significantly increased when PBMC were stimulated with R848 compared to untreated PMBC (*p* = 0.0001, *p* = 0.0288, respectively). In the CD10+ cells, the expression levels of HLA-I significantly increased when they were stimulated with imiquimod (*p* = 0.0436) and ODN (*p* = 0.0436) (Figure 8C) compared to untreated PMBC. Meanwhile, the proportion of CD19+ (Figure 8D) and CD19+CD10+ (Figure 8E) cells that expressed MHC-I did not change, and the proportion of CD10+ cells that expressed HLA-I significantly increased only with R848 (*p* = 0.0021) compared to untreated PBMC (Figure 8F).

### 2.8. TLR8 and TLR9 Ligands Increase the Cytotoxic Activity of NK Cells

**Figure 9:**
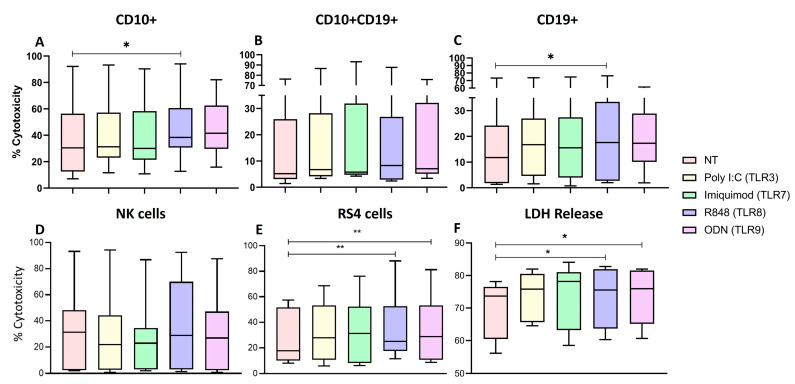
Cytotoxic activity of PBMCs and NK cells. (**A**) The median of the percentage of CD10+ blast cells was determined for each treatment and compared with the percentage of non-treated cell death. (**B**) The median of the percentage of CD10+CD19+ blast cells was determined for each treatment and compared with the percentage of non-treated cell death. (**C**) The median of the percentage of CD19+ blast cells was determined for each treatment and compared with the percentage of non-treated cell death. (**D**) NK cell death was determined for each treatment and compared with the percentage of non-treated cell death. (**E**) The median of the percentage of RS4 death was determined for each treatment and compared with the percentage of non-treated cell death. (**F**) Cell-mediated cytotoxic activity, the median of the percentage of LDH released into the supernatant of cocultures of RS4 cells with NK cells. The box and whisker plot was employed to visualize the distribution of the data, highlighting the central tendency, spread and the outlier’s values within the dataset. The Friedman test was used for the comparison. * *p* < 0.05, ** *p* < 0.005.

To evaluate if the functional and phenotypic changes of the NK cells previously observed affected their antitumoral activity against leukemic cells, two cytotoxicity assays were performed (Figure 9).

We measured the viability of leukemic blasts (CD10+, CD10+CD19+ and CD19+), NK cells and leukemic RS4; 11 cells in co-culture using Zombie NIR (viability marker) to determine if the blast lysis capacity was increased in the NK cells treated with the four TLRs ligands.

We observed that in the case of CD10+ leukemic blasts, there was a significant increase in cytotoxicity when they were treated with TLR8 ligand, R848 (Figure 9A) (*p* = 0.0288), and that none of the ligands caused cytotoxicity on the CD10+CD19+ blasts (Figure 9B) and in the case of CD19+ blast there was a significant increase in cytotoxicity when they were treated with TLR8 ligand, R848 (Figure 9C) (*p* = 0.0233).

We Also observed that with all the treatments the median of the proportion of dead NK cells decreased (Poly I:C = 21.80, Imiquimod = 22.90, R848 = 28.80, and ODN = 26.90) when compared with the non-treated cells (M = 31.20) (Figure 9D). Interestingly, we observed that R848 and ODN2006 significantly increased the cytotoxicity against the leukemic RS4 cells (*p* = 0.0162 and *p* = 0.0071, respectively) (Figure 9E).

To further confirm the cytotoxicity of the NK cells isolated from the ALL patients towards the RS4; 11, we measured LDH released in the supernatant from the RS4 co-culture with stimulated NK cells. We calculated the % of cytotoxicity of the target cells. Interestingly, R848 and ODN2006 significantly increased the % of cytotoxicity against the leukemic RS4; 11 cells (*p* = 0.0325 and *p* = 0.0186, respectively) (Figure 9F).

All of these results together suggest that stimulation with R848 and ODN may increase the cytotoxicity of the NK cells from ALL patients against the leukemic cells.

## 3. Discussion

This study aimed to evaluate the response of NK cell subpopulations from pediatric patients with ALL to endosomal TLR ligand stimulation and compare them with those of the unstimulated NK cells from the same patients, with the objective of identifying the feasibility of activating the NK cells from ALL patients and the most suitable molecule(s) with potential as adjuvants in combination with cancer immunotherapy. We show that the agonists poly I:C, imiquimod, R848, and ODN2006 increase the function of NK cells, with R848 and ODN increasing the cytotoxicity. In addition, our study showed that the most sensitive NK subpopulation was the CD56+CD16−, which is considered mainly immunoregulatory, suggesting that this subpopulation may be also contributing to blast lysis.

Existing studies performed on healthy donor cells and solid tumors have shown that NK cells can be directly activated by TLR agonist stimuli, increasing their immunoregulatory and cytotoxic activities in that specific context [32,33] leaving uncertainties about the response patterns in hematological malignancies. In cancer situations, including ALL, exposure to tumor cells and the tumor microenvironment components hinders NK cell functions, rendering them dysfunctional [9,34]. Given that NK cells in pediatric patients with ALL are already influenced by disease [12], with our study design, we aim to assess the behavior of NK cells following stimulation with specific endosomal TLR in both PBMCs and in isolation in order to investigate whether the known TLR activation observed in healthy donors is feasible both within their natural microenvironment and when isolated or is already affected in the pediatric leukemia population. This is the first study to assess the response of different NK subpopulations from ALL patients after stimulation with TLR’s ligands, providing valuable understanding into the functional characteristics of NK cells in pediatric hematological malignancies.

The finding that Poly I:C and R848 notably increased the IFN-γ positive cells in the CD56+CD16− subpopulation within the PBMCs and that the four TLR ligands upregulated the IFN-γ production in the CD56−CD16+ subpopulation of isolated NK cells suggests the potential of TLR agonists to enhance NK cell-mediated immunoregulatory responses, since IFN-γ is a key cytokine involved in the immune response against infections and tumors, all in the context of ALL. The fact that these responses can be observed in the immunoregulatory and cytotoxic subsets suggests a broad impact from the TLR ligands on NK cell immune activity, encompassing both subsets. These results align with the evidence on healthy donors that TLR agonists can increase the production of IFN-γ by NK cells [35].

Another characteristic of functional NK cells is the release of cytotoxic granules such as perforin and granzyme B, this degranulation is measured by the expression of CD107a on the cell membrane. The increased expression of this molecule indicates the cytotoxic activity of the NK cell. In our study, Poly I:C, ODN, and R848 were shown to have a potential impact on the cytotoxic effect of NK cells since they increased NK cells’ CD107 expression and percent within the PBMCs. On the other hand, the results on isolated NK cells showed that Imiquimod (TLR7) modified the expression and proportion of cells expressing CD107a for CD56+CD16− [36,37].

The distinct responses observed in PBMCs versus isolated NK cells highlight the influence of the cellular microenvironment on NK cell activation [26]. Stimulation within the PBMC milieu may involve interactions with other immune cells, potentially amplifying the response to TLR agonists [24]. Conversely, isolated NK cells may respond more directly to TLR stimulation, providing insights into the intrinsic activation mechanisms of these cells. Further studies are warranted to elucidate the precise signaling pathways involved in TLR-mediated NK cell activation and to explore the potential synergistic effects of combining different TLR agonists.

To further evaluate the degranulation on the isolated NK cells, we evaluated the concentration of Granzyme B on the supernatant, for this enzyme is one contained in the cytotoxic granules of NK cells [38]. We observed that ODN was the one that stimulated the secretion of Granzyme B from these cells, which is in odds with our finding regarding the expression of CD107a which increased with Imiquimod. Therefore, this should be further studied to fully understand the phenomenon.

It has been suggested that TLR7/8 activation promotes tumors by inducing robust pro-inflammatory cytokine secretion and activating NK at the tumor site [33]. IL-1β and IL-2 have been suggested as a potent co-stimulus for IFN-γ production by the immunoregulatory NK cells [36,39]. TNF-α, IFN-γ, and CXCL8 are produced by NK cells and these cells also enhance their activation and recruitment [40,41]. Concordantly, we observed that R848 increased these proinflammatory cytokines on the supernatant of PBMCs. These results suggest that monocytes, T cells, and dendritic cells are stimulated and subsequently may promote the activation of NK cells which could explain the increase in IFN-γ and CD107a as observed on the CD56+CD16− subpopulation.

NKG2D is an activating receptor that is expressed in NK cells independently of the activation status of the cell. However, since the activation of NK cells depends on the balance between the activator and inhibitor receptors [42] an increase in the expression of this receptor may lead to the polarization of the NK cells to activated status. Therefore, our findings that Imiquimod and ODN increased the expression of the NKG2D in the cytotoxic subpopulation within the PBMCs suggest that these ligands may help the NK cells to their cytotoxic status. Conversely, the upregulation of NKG2D expression by Imiquimod R848 and Poly I:C in the isolated NK cells suggests a potential role in enhancing NK cell cytotoxicity against ALL cells. This aligns with previous studies indicating that NKG2D is involved in the recognition and elimination of tumor cells [43]. However, further investigation is warranted to elucidate the specific mechanisms underlying TLR-mediated NKG2D upregulation and its functional consequences in pediatric ALL.

The other receptor, NKp44, is associated with an activated state of the NK cells [44]. An increase in NKp44 expression and the number of cells expressing it may suggest an improvement in NK cell functionality. Our study found that Poly I:C, R848, and ODN showed an activation potential over NK cells from patients with ALL by increasing the expression of this receptor and its proportion both within PBMCs and isolated NK cells. The findings from our study suggest that Poly I:C, R848, and ODN may restore effectively the activation capacity of NK cells of ALL patients since the observed increase in NKp44 expression could reflect a heightened state of NK cell activation, potentially leading to improved cytotoxicity against tumor cells. Previous research has shown that NKp44 is involved in the recognition and targeting of tumor cells, indicating its importance in antitumor immunity [44].

The different responses to TLR of the NK cells embedded in PBMC versus the isolated NK cells in our study confirm the microenvironment cells’ influence on NK responses in addition to the TLR stimulation [34] and should be further evaluated. However, in both aspects, CD56+CD16− was the most sensible to the stimulation, suggesting that this subpopulation could be less impaired and its potential for a therapeutic approach should be further analyzed.

Some HLA-I molecules may play a role as ligands for activating NK receptors, such as NKG2D [43]. The stimulation with the TLR8 ligand (R848) significantly increased the expression of HLA-I on the CD19+ B lymphocytes and CD19+CD10 blast subpopulation, while Imiquimod (TLR7) and ODN (TLR9) increased it on all CD10+ cells. These results are relevant since knowing the nature of these HLA-I (inhibitory o activating ligands) may help us to understand the answer that NK cells will elicit.

The assessment of our ALL-patients NK cell cytotoxicity is crucial for understanding the antitumor efficacy of endosomal TLR ligands in the context of ALL. In our study, we observed that in the context of the PBMC, R848 increased the cytotoxic activity of cells from patients with ALL against their Leukemic blasts this finding is a positive approach since the activation of PBMC reflects a microenvironment context, therefore this cytotoxic activity against the patient blast suggests that R848 has potential as a cell activation in the context of hematological malignancies.

The observation that R848 and ODN significantly increased the cytotoxic activity of NK cells from patients with ALL against the RS4 cell line helps to further confirm our first finding. This enhancement in cytotoxic activity suggests that these TLR agonists have the potential to improve the ability of NK cells from ALL patients to recognize and eliminate leukemic cells. Interestingly, our findings regarding Granzyme B secretion align with these results since R848 and ODN also increase this granule release, indicating that Granzyme B may indeed play a role in mediating blast lysis. However, it is important to note that while our results suggest a potential mechanism for the observed increase in cytotoxicity of these ALL-NK cells, further investigation is needed to confirm this hypothesis. Additional studies, such as blocking experiments, could help elucidate the specific role of Granzyme B in TLR-mediated enhancement of NK cell cytotoxicity.

Furthermore, our findings are similar to previous research by Veziani et al. [39], who demonstrated that specific TLR8 agonists increase cytokine production and cytotoxic activity in NK cells from healthy donors. This alignment with existing literature helps us to start to elucidate that is feasible to activate and enhance the functions of these leukemic NK cells through TLR agonists in the context of hematological malignancies.

These findings highlight the potential of TLR agonists, particularly TLR8 agonists like R848, as promising candidates for immunotherapeutic strategies in pediatric ALL. By enhancing NK cell cytotoxicity and cytokine production, TLR agonists could offer a novel approach to augmenting the antitumor immune response in pediatric patients with ALL. However, further studies, including in vivo models and clinical trials, are needed to validate these findings and determine the feasibility of incorporating TLR agonists into therapeutic regimens for pediatric ALL.

In our work, it was evident that when the isolated CD56−CD16+ were stimulated, the increase in degranulation was not present with none of the TLR ligands, and this is in concordance with the literature that this subpopulation is “dysfunctional” due to the reduced cytotoxic potential, notably against tumor target cells [45]. Recently, this population has been described in Kenyan children with Burkitt lymphoma [46] and in non-virally Acute Lymphoblastic Leukemia patients [47] with adverse clinical outcomes. A proteomic analysis showed that CD56−CD16+ shared some similarities with CD56dimCD16+ NK cells [48], supporting the classification of this subset as NK cells. Furthermore, it has been suggested that this phenotype with a CD56 downregulated emerges in the presence of an immunosuppressive milieu [49], even when the mechanism by which this happens remains unknown. This may explain the presence of this subpopulation in our patients with ALL and the impaired (lack of) function even after the stimulation. In our study we reported CD56−CD16+ as NK cells when isolated since we used negative selection, assuring the elimination of non-NK cells (CD14+, CD11c) and in de PBMCs due to the expression of NKG2D in 90% of this subpopulation.

Our results support the hypothesis that the activation with TLR ligand may restore functionality and promote the expression of activation receptors of NK cells from patients with ALL. Various clinical trials with these studied ligands in solid tumors lay the groundwork for a potential translation to clinical applications in hematological malignancies. Poly I:C has been studied in combination with anti-PD1 on solid tumors (NCT03721679) [50] and in Subjects with Unresectable Hepatocellular Carcinoma (NCT03732547) [51]. Some Imiquimod trials evaluated effectiveness in cutaneous metastases of melanoma (NCT01264731) [52] and cervical high-grade squamous intraepithelial neoplasia (NCT05405270) [53]. One clinical trial for R848 evaluated the effect on T-cells in cutaneous T-cell lymphoma (NCT01676831) [54] and as an adjuvant with vaccine therapy for melanoma (NCT00470379) [55]. ODN has been studied as an Injection Booster against solid tumors (NCT04952272) [56].

We acknowledge the limitations of our study confined to in vitro scenarios. Nonetheless, the significance of this information in literature serves as a crucial precedent. It indicates that NK cells, known to exhibit compromised functionality in hematologic neoplasms, possess the capacity to restore and enhance their cytotoxic and immunoregulatory anti-tumor functions through their endosomal TLR receptor stimulation. This sets the stage for a promising avenue of research, suggesting that these molecules warrant further exploration with an immunotherapeutic approach against hematologic neoplasms.

## 4. Materials and Methods

### 4.1. Patients

Twenty-four pediatric patients newly diagnosed between March 2022 and July 2023 with ALL, were included. The inclusion criteria involved: Children aged 1 to 18 years, recently diagnosed with acute lymphoblastic leukemia (ALL), who had not received prior treatment, and who had not undergone antibiotics or transfusions and participants and their parent or guardian who provided informed consent. The exclusion criteria included patients with other hematological disorders or malignancies, those with infections or immunodeficiencies, and individuals who did not provide consent. Peripheral blood samples were taken with the prior informed consent of the patient’s parents or guardians, in accordance with institutional protocols.

This study was approved by the Research, Ethics, and Biosafety Committees of the Hospital Infantil de México Federico Gómez, following the international guidelines for biomedical research in humans (approval no. CIOMS-WHO 1993).

### 4.2. Obtaining Mononuclear Cells, Isolating NK Cells, Culture and Activation with TLR Ligands

Mononuclear cells were isolated from peripheral blood by density gradient centrifugation with Lymphoprep™ (Axis-Shield, Boston, MA, USA), according to the manufacturer’s protocol. To assess the direct activation of NK cells through TLRs, they were enriched from peripheral blood mononuclear cells using an EasySep™ Human NK Cell Isolation Kit (STEMCELL Technologies Inc., Vancouver, BC, Canada), according to the manufacturer’s protocol.

The obtained mononuclear cells (1 × 10^6^) were cultured in 1 mL of RPMI medium supplemented with 2% fetal bovine serum (FBS), in 48-well plates, and the enriched NK cells (1 × 10^5^) were cultured in 0.5 mL of RPMI medium supplemented with 2% FBS. The TLR ligands used, and their doses were: poly:IC 1 μg/mL (Sigma-Aldrich, St. Louis, MO, USA), Imiquimod 0.1 μg/mL (Enzo Life Science, Plymouth, PA, USA), R-848 5 μg/mL (Enzo Life Science, Plymouth, PA, USA), and ODN 2006 1 ng/mL (Invivo, San Diego, CA, USA) (all concentrations were previously determined). Stimulation was left to proceed at 37 °C in a 5% CO_2_ atmosphere for 24 h.

### 4.3. Characterization of NK Cells and Determination of Activation

To characterize NK cells, they were stained for 20 min at 4 °C, with 20 μL of staining mix containing staining buffer (phosphate-buffered saline (PBS) with 1% serum albumin) and the following antibodies antihuman: anti-CD3-pacific blue, (0.2 μg per 10^6^ cells) anti-CD56-APC, (0.5 μg per 10^6^ cells) anti-CD19-PECy7 (0.5 μg per 10^6^ cells) (All of them from BD Biosciences, San Jose, CA, USA), and anti-CD16-PerCPcy5.5 (2.5 μL per 10^6^ cells) (BioLegend, San Diego, CA, USA). To determine the activation of NK cells, the change in the expression of CD107a and intracellular IFN-γ was used, as well as the expression of receptors NKG2D and NKp44; the expression of HLA class I was also determined. The cells were stained with anti-CD107a-FITC, (2.5 μL per 10^6^ cells) anti-HLA ABC-PE, (5 μL per 10^6^ cells) anti-NKG2D-APCCY7 (2.5 μL per 10^6^ cells) (BioLegend, San Diego, CA, USA), and anti-NKp44-VioBrightFITC (1 μg per 10^6^ cells (Merck Millipore, Burlington, MA, USA)). For intracellular staining, monensin (2 μM) (Biolegend, San Diego, CA, USA) was added to the culture during the last 4 h of treatment. To detect intracellular cytokine production, cells were fixed, permeabilized with CytoFix/Cytoperm (BD Bioscience) following the manufacturer’s protocol and stained with anti-IFN-γ-PECy7 (2.5 μL per 10^6^ cells (Abcam, Cambridge, UK) according to manufacturer’s instructions. The maximum staining mix volume used for the tube of treatment was 20 μL. The staining buffer volume varied depending on the tubes to stain.

Reading was performed on the CytoFLEX flow cytometer (Beckman Coulter, Brea, CA, USA) and analysis was performed with the FlowJo software V.10.

### 4.4. Granzyme B Detection

The concentration of granzyme B in the supernatants of cells cultured and treated with TLR ligands was determined using the commercial Human Granzyme B DuoSet ELISA kit (R&D Systems, Minneapolis, MN, USA), following the manufacturer’s instructions. The optical density of each well was measured at 450 and 570 nm in a microplate reader (Agilent BioTek™ Epoch, Santa Clara, CA, USA) with a margin of no more than 30 min.

### 4.5. Cytokines Determination

The concentration of cytokines (IFN-γ, IL-2, IL-1β, TNF-α, and IL-6) and chemokines (IL-8 and MCP-1) in the supernatants of cells cultured and treated with TLR ligands was determined using the commercial kit Human Cytokine/Chemokine Magnetic Bead Panel-Immunology Multiplex Assay (Millipore, Burlington, MA, USA), following the manufacturer’s instructions. The optical density of each well was measured with the MagPix plate reader (Luminex, Austin, TX, USA).

### 4.6. Interferon Gamma Determination

IFN-γ was determined in supernatants of enriched NK cells. After 24 h of stimulation, of the enriched NK cells, with the TLRs ligands, the supernatant was collected and stored at −20 °C in aliquots until analysis. The sample was thawed only once to avoid degradation. IFN-γ was determined with the commercial ELISA IFN-γ OptEIA kit (BD Biosciences, San Diego, CA, USA), according to the manufacturer’s instructions. The optical density of each well was measured at 450 nm in a microplate reader (Agilent BioTek™ Epoch, Santa Clara, CA, USA) with a margin of no more than 30 min.

### 4.7. Cytotoxicity Assays

#### 4.7.1. Zombie NIR Assay

The increase in cytotoxic activity of NK cells stimulated with the specific TLR ligands was measured by using a viability marker (Zombie NIR, Biolegend, San Diego, CA, USA). Briefly: enriched NK cells from patients with leukemia were co-cultured with the RS4;11 CRL-1873 cell lines from ATCC in a 1:2 ratio in RPMI medium with 2% FBS; then, they were stimulated or not with each ligand of TLR3, TLR7, TLR8, and TLR9 and incubated for 24 h at 37 °C with 5% CO_2_. After incubation, cells were harvested and stained with 100 μL of Zombie NIR (1:1000 dilution with PBS) to evaluate viability in a CytoFLEX flow cytometer (Beckman Coulter). Analysis was performed with the FlowJo software version V.10. We also harvested the PBMC cells (NKs and leukaemic cells) after 24 h activation with the specific ligand and stained with 100 μL of Zombie NIR (1:1000 dilution with PBS) to evaluate the viability of the blasts.

#### 4.7.2. LDH Release Assay

This assay was also used to measure the cytotoxicity of the effector cells (enriched NK cells from ALL patients) against the target cells (RS4;11 CRL-1873 cell line from ATCC). We used the commercial kit CytoTox 96 non-radioactive assay (Promega, Madison, WI, USA) to measure the concentration of LDH released by the dead target cells after the co-culture with the NK cells, following the manufacturer’s instructions. Briefly: stimulated enriched NK cells were co-cultured with the RS4 cell line in RPMI medium with 2% FBS, for 24 h at 37 °C with 5% CO_2_. After incubation, they were centrifuged and the supernatant was stored at −20 until used. The supernatant of co-cultures with each treatment and the supernatant of NK cells and RS4 cell lines alone were placed in triplicate in a 96-well plate. The substrate provided by the kit was added to each well and the plate was incubated for 30 min at 37 °C protected from light. Finally, a stop solution was added and reading was carried out at 490 nm in a microplate reader (Agilent BioTek™ Epoch, Santa Clara, CA, USA) with a margin of no more than 30 min. To determine the cytotoxic activity of the NK cells against target RS4; 11 cells, we used the following formula.

$$\%\;Cytotoxicity=\frac{Experimental\;OD-Effector\;Spontaneous\;OD-Target\;spontaneous\;OD}{Target\;Maximum\;OD-Target\;Spontaneous\;OD}\times 100$$

### 4.8. Statistical Analysis

The median frequencies of the different subpopulations of peripheral blood NK cells and enriched NK cells from patients with ALL from each treatment were compared with the non-treated cells using a Friedman test. GraphPad Prism version 8 (GraphPad Software Inc., Boston, MA, USA) was used for the statistical analysis.

## 5. Conclusions

It is possible to directly activate the NK cells from ALL by endosomal TLRs. This activation promotes a different behavior from the different NK cell subpopulations than when activated within the PBMCs. The activation via endosomal TLRs increases the functional activity of the NK cells in ALL patients. Furthermore, direct activation of the NK cells via TLR8 and TLR9 with R848 and ODN respectively increased the anti-tumoral activity of the NK cells, since they increased the blast lysis. These findings provide a foundation for further exploration of TLR ligands as potential immunotherapeutic agents to enhance NK cell-mediated anti-tumor responses in ALL.

## Figures and Tables

**Table 1:** Demographic and genetic characteristics of the study group. Patients with acute lymphoblastic leukemia.

Characteristics (Peripheral blood)	
N° of cases	24
Sex M:F	14:10
Age media (range)	7 (1–17) years
Immunophenotype	
Pre-B	22
not defined	2
Chromosomic alterations	
SNP TPMT Heterozygous	4
Translocation (12:21)	2
Translocation (2:3)	1
Translocation (9:22)	2
Translocation (1:19)	2
Without translocations	6
Not defined	7
% NK Cells (range)	1.87 (0.4–5.97)

M: Male, F: Female; NK: Natural Killer; SNP: Single nucleotide polimofism; TPMT: thiopurine S-methyltransferase.

## Data Availability

The datasets during the current study are available from the corresponding author upon reasonable request.

## Supplementary Materials

The following supporting information can be downloaded at: https://www.mdpi.com/article/10.3390/ijms25137500/s1.
