# Supplementary material for: TLR Agonists Modify NK Cell Activation and Increase Its Cytotoxicity in Acute Lymphoblastic Leukemia
Source: Int J Mol Sci. 2024 Jul 8;25(13):7500. doi: 10.3390/ijms25137500 (PMC11242025; doi:10.3390/ijms25137500)
Supplement: Supplementary file 1 [file ijms-25-07500-s001.zip › ijms-3000479-supplementary.pdf]

Supplementary Material

1. **Supplementary Figures**

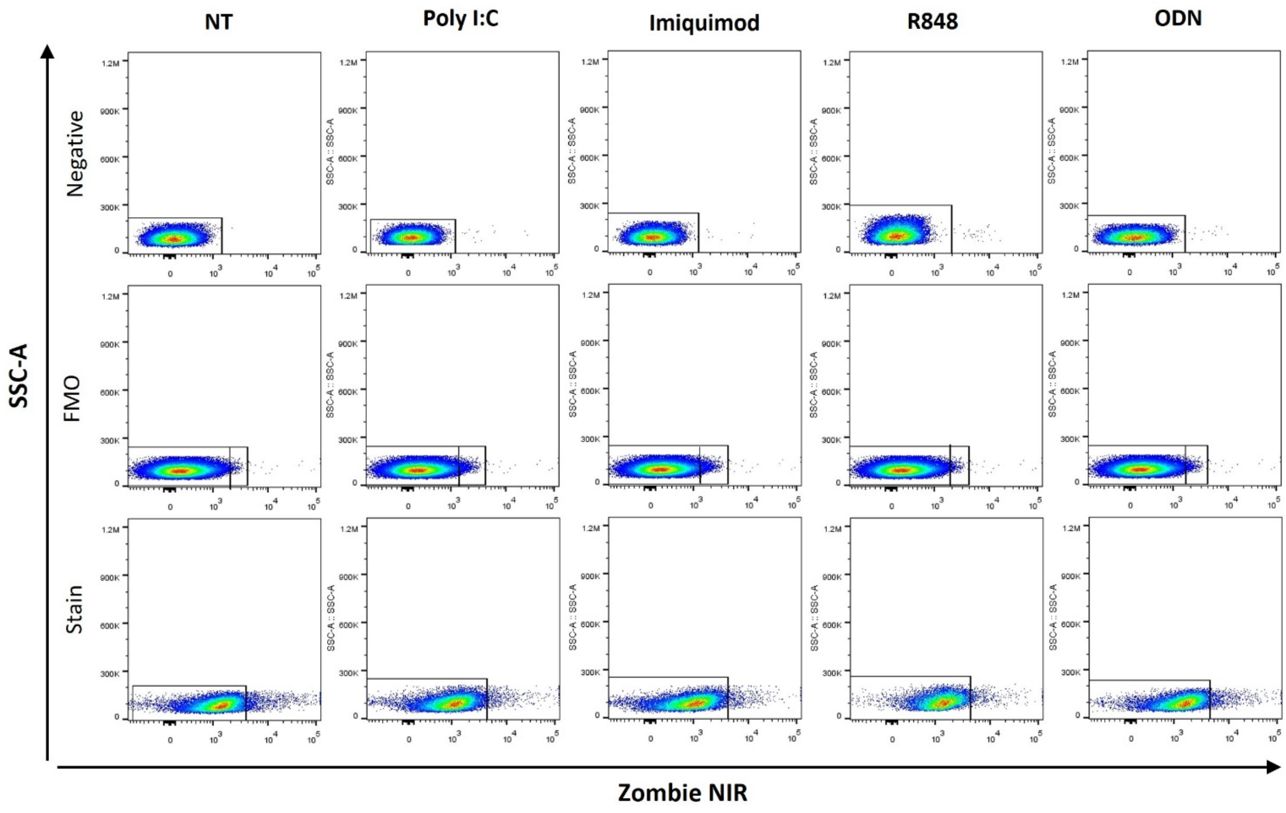

Figure S1. Viability Cytometry. For each sample analyzed through cytometry, the inclusion of a viability marker (Zombie NIR-APCCy7) was integral. This marker served the dual purpose of assessing cell viability at the time of acquisition and 24 hours post-treatment. Additionally, its incorporation was essential to prevent contamination in each gate, mitigating the risk of non-specific antibody binding to deceased cells.

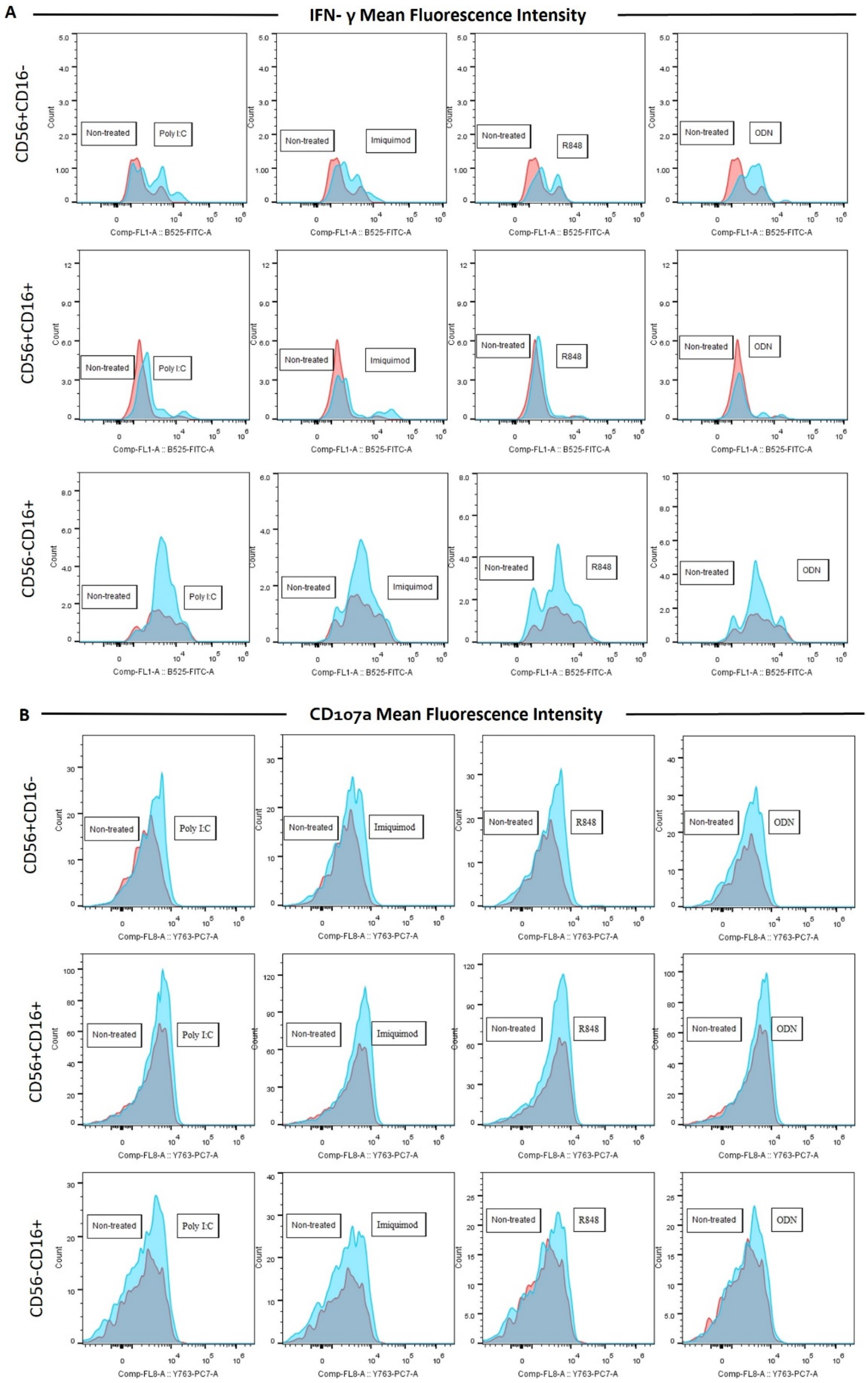

Figure S2. MFI of Activation markers of the NK cells within PBMCs after endosomal TLR stimulation. Mean Fluorescence Intensity of IFN-γ and CD107a expressed in the different NK subpopulations after each treatment vs non-treated cells.
